# Complete mitogenomes of *Anopheles peditaeniatus* and *Anopheles nitidus* and phylogenetic relationships within the genus *Anopheles* inferred from mitogenomes

**DOI:** 10.1186/s13071-021-04963-4

**Published:** 2021-09-06

**Authors:** Jing Guo, Zhen-Tian Yan, Wen-Bo Fu, Huan Yuan, Xu-Dong Li, Bin Chen

**Affiliations:** Chongqing Key Laboratory of Vector Insects, Institute of Entomology and Molecular Biology, College of Life Sciences, Chongqing Normal University, Chongqing, 401331 People’s Republic of China

**Keywords:** Mitogenomes, Phylogenetics, Culicidae, *Anopheles*, *Anopheles peditaeniatus*, *An. nitidus*

## Abstract

**Background:**

Despite the medical importance of mosquitoes of the genus *Anopheles* in the transmission of malaria and other human diseases, its phylogenetic relationships are not settled, and the characteristics of mitochondrial genome (mitogenome) are not thoroughly understood.

**Methods:**

The present study sequenced and analyzed the complete mitogenomes of *An. peditaeniatus* and *An. nitidus*, investigated genome characteristics, and inferred the phylogenetic relationships of 76 *Anopheles* spp.

**Results:**

The complete mitogenomes of *An. peditaeniatus* and *An. nitidus* are 15,416 and 15,418 bp long, respectively, and both include 13 PCGs, 22 tRNAs, two tRNAs and one control region (CR). Mitogenomes of *Anopheles* spp. are similar to those of other insects in general characteristics; however, the *trnR* and *trnA* have been reversed to “*trnR*-*trnA*,” as has been reported in other mosquito genera. Genome variations mainly occur in CR length (493–886 bp) with six repeat unit types identified for the first time that demonstrate an evolutionary signal. The subgenera *Lophopodomyia*, *Stethomyia*, *Kerteszia*, *Nyssorhynchus*, *Anopheles* and *Cellia* are inferred to be monophyletic, and the phylogenetic analyses support a new phylogenetic relationship among the six subgenera investigated, in that subgenus *Lophopodomyia* is the sister to all other five subgenera, and the remaining five subgenera are divided into two clades, one of which is a sister-taxon subgenera *Stethomyia* + *Kerteszia*, and the other consists of subgenus *Nyssorhynchus* as the sister to a sister-group subgenera *Anopheles* + *Cellia*. Four series (Neomyzomyia, Pyretophorus, Neocellia and Myzomyia) of the subgenus *Cellia*, and two series (Arribalzagia and Myzorhynchus) of the subgenus *Anopheles* were found to be monophyletic, whereas three sections (Myzorhynchella, Argyritarsis and Albimanus) and their subdivisions of the subgenus *Nyssorhynchus* were polyphyletic or paraphyletic.

**Conclusions:**

The study comprehensively uncovered the characteristics of mitogenome and the phylogenetics based on mitogenomes in the genus *Anopheles*, and provided information for further study on the mitogenomes, phylogenetics and taxonomic revision of the genus.

**Graphical abstract:**

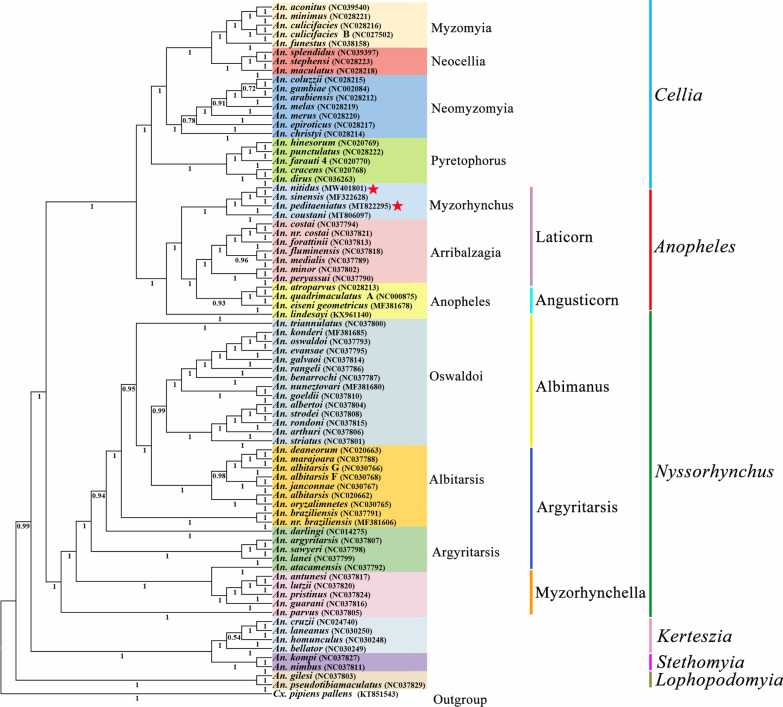

**Supplementary Information:**

The online version contains supplementary material available at 10.1186/s13071-021-04963-4.

## Background

The genus *Anopheles* belongs to the subfamily Anophelinae in Culicidae (mosquitoes). It is the most diverse genus in the subfamily, with 475 formally named species and more than 50 unnamed members of species complexes worldwide [1]. Anophelinae mosquitoes can transmit a variety of diseases, and are one of the most important groups of insects in medicine, as they are the unique vectors of human malarial parasites, which caused 229 million cases and 409,000 deaths worldwide in 2019 [2]. In addition to malaria parasites, mosquitoes in *Anopheles* also transmit filarial parasites [3]. Some studies have shown that *Anopheles* mosquitoes also harbor arboviruses, which multiply in the mosquito vectors before transmission to a vertebrate host, such as o’nyong-nyong [4]. Due to their exceeding importance, mosquitoes of this genus are subject to more taxonomic studies than any other mosquito group.

The classification of *Anopheles* started more than 100 years ago [5], when it was treated as one of 18 genera in the Anophelinae, while *Cellia*, *Nyssorhynchus*, *Stethomyia* and *Kerteszia* were also treated as independent genera based on morphological characteristics. Subsequently, the five genera were successively included as subgenera of the genus *Anopheles* based on the number and location of specialized setae on the male genital gonocoxites and other characteristics [6–8]. Three additional subgenera, *Lophopodomyia*, *Baimaia* and *Christya* were established within the genus *Anopheles* [9–11]. Due to the diversity of species contained in the subgenera *Anopheles*, *Cellia* and *Nyssorhynchus*, taxonomists divided some species into informal categories such as sections, series and groups. The earliest phylogenetic studies for *Anopheles* were mainly based on morphological characters and single genes. Different data sets and phylogenetic inference methods often lead to inconsistent results between studies, and therefore phylogenetic relationships in *Anopheles* have not been well settled.

There have been a number of representative phylogenetic studies on the genus *Anopheles*. An analysis including 63 species in Anophelinae based on 163 morphological characters suggested the monophyly of the subgenera *Cellia*, *Nyssorhynchus*, *Stethomyia*, *Kerteszia* and *Lophopodomyia* [12]. In *Nyssorhynchus*, the three sections Albimanus, Argyritarsis and Myzorhynchella were suggested to be paraphyletic. In *Cellia*, only the series Cellia was considered to be monophyletic. In *Anopheles*, series Arribalzagia and Lophoscelomyia were considered to be monophyletic, while the series Cycloleppteron + Arribalzagia was nested within series Myzorhynchus [12]. Some further morphology-based studies also suggested the monophyly of the subgenera *Nyssorhynchus*, *Cellia* and *Kerteszia*, and displayed the sister relationship between subgenera *Kerteszia* and *Nyssorhynchus* [11, 13, 14]. An analysis based on *COX1* + ITS2 dataset suggested the monophyly of subgenera *Anopheles* and *Cellia*, and the analysis using ITS2 dataset alone resulted in the same conclusion, which was not supported by the *COX1* dataset alone [15]. Two studies based on the mitogenomes, including 50 and 33 species, respectively, both also supported the monophyly of the subgenera *Anopheles*, *Nyssorhynchus*, *Cellia* and *Kerteszia* [16, 17]. Generally, the monophyly of the subgenera *Anopheles*, *Nyssorhynchus*, *Cellia*, *Stethomyia*, *Kerteszia* and *Lophopodomyia* has been supported by most recent studies; however, sections and series within the subgenera *Anopheles*, *Nyssorhynchus* and *Cellia* have not been well resorted. There is a need to elucidate the phylogeny of the genus *Anopheles* using more species, more data and updated phylogenetic analysis approaches.

The mitochondrion is an important organelle in eukaryotic cells, with a genome independent of the nucleus, the mitochondrial genome (mitogenome) [18]. The mitogenome typically has a small genome size, low levels of recombination and maternal inheritance, and therefore it has been widely used as a molecular marker for the identification of species, phylogenetic inference and population structure research [19, 20]. Since the publication of the first insect mitogenome (*Drosophila yakuba*) in 1985 [21], the number of insect mitogenomes have increased rapidly. Phylogenetic studies based on insect mitogenomes have shown good results in Diptera [22], Orthoptera [23], Coleoptera [24] and Hymenoptera [25]. To date the complete mitogenomes of 125 species of Culicidae have been sequenced, of which 74 species are from the genus *Anopheles*. Dipteran mitogenomes are mostly 14–20 kb long, including 37 genes—13 protein-coding genes (PCGs), two ribosomal RNA (rRNA) genes, 22 transfer RNA (tRNA) genes and a control region (CR)—and these genes are arranged in a compact circular genome [26]. The genome structure in all reported mosquito mitogenomes is similar to the typical mitogenomes of Diptera; however, the *trnA* and *trnR* of mosquitoes are rearranged to form “*trnR*-*trnA*” [16, 17, 21].

In the present study, we sequenced and annotated the complete mitogenomes of *An. peditaeniatus* and *An. nitidus*, and analyzed the mitogenome characteristics of 76 species in the genus *Anopheles*. Additionally, we constructed the phylogenetic relationships of these 76 species. This study provides new insights into the mitogenome characteristics and phylogenetic relationships in the genus *Anopheles*.

## Methods

### Sample collection and DNA extraction

Specimens of *An. peditaeniatus* and *An. nitidus* were collected from Yadong County (29° 11′ 46″ N, 95° 12′ 11″ E), Tibet, China, in July 2014, and Tiebei County, Jilin Province, China (42° 27′ 21″ N, 128° 06′ 18″ E) in July 2013. All samples were preserved in individual vials. After morphological identification using keys reported previously [27], samples were stored in 100% alcohol and housed at −20 °C until DNA extraction. Total DNA was extracted from an individual adult mosquito using the QIAGEN Genomic DNA Kit [28], and used for 350 bp library construction and Illumina high-throughput sequencing by Shenzhen Huitong Biotechnology Co. Ltd.

### Mitogenome sequencing annotation and characteristics analysis

Genome sequencing using paired-end sequencing (PE 150) was carried out using the Illumina HiSeq X Ten platform by Huitong Biotechnology Co., Ltd. In total, 20.41 Gb (*An. peditaeniatus*) and 25.96 Gb (*An. nitidus*) clean data were obtained after filtering of raw data (20.54 Gb for *An. peditaeniatus* and 26.15 Gb for *An. nitidus*) using the NGS QC Toolkit [29], and the sequencing depth was 288.9X (*An. peditaeniatus*) and 5162X (*An. nitidus*). Subsequently, the mitogenome reads were extracted using the BLAST program with *An. sinensis* mitogenome sequence as reference, and assembled using de novo mitogenome assembly with SPAdes 3.9.0 [30].

The mitogenomes of *An. peditaeniatus* and *An. nitidus* were annotated using MITOS (http://mitos.bioinf.unileipzig.de/index.py) [31]. Protein-coding gene and ribosomal RNA gene annotations were confirmed by reference to published mosquito mitogenomes and corrected in Geneious v4.8.5 [32]. The secondary structures of tRNAs were predicted using MITOS [31], and the structure maps of the mitogenomes were visualized using OGDRAW1.3.1 [33]. Base composition, codon usage, relative synonymous codon usage (RSCU) and amino acid content were computed with MEGA v.7.0.26 [34]. Nucleotide compositional bias was calculated using the formulas AT-skew = [A − T]/[A + T] and GC-skew = [G − C]/[G + C] [35], and three-dimensional scatter plots of AT-skew, GC-skew and AT% were drawn using OriginPro v.9.0 [36]. Selection pressure on the 13 PCGs was analyzed by calculating Ka and Ks values using DnaSP v6.12.03 [37]. Sequence motifs in the CR were identified using Tandem Repeats Finder [38].

### Phylogenetic analysis

Multiple sequence alignments of the PCGs were performed on the TranslatorX server (http://translatorx.co.uk/) using the MAFFT amino acid alignment mode. Gblocks with the default setting in TranslatorX was used to remove the ambiguously aligned positions. Individual alignments were concatenated in SequenceMatrix [39]. PartitionFinder 2.0 was used to determine the best-fit substitution model for each gene according to the Akaike information criterion (AIC), and the default values for the initial partition settings were applied [40]. Phylogenetic analyses were performed using maximum likelihood (ML) inference in IQ-TREE 1.6.10 [41] and Bayesian inference (BI) analysis in MrBayes v.3.2.7a [42] using *Culex pipiens pallens* as outgroup (Table 1). Bootstrap values were calculated using 1000 replicates for ML. BI was performed as two independent runs, each with four chains, and these chains ran simultaneously for 10,000,000 generations, with sampling every 1000 steps, and a 25% burn-in rate. Phylogenetic trees were drawn using FigTree v.1.4.4 (http://tree.bio.ed.ac.uk/software/figtree/).

**Table 1:** Detailed sequence information of mitochondrial genomes used in the present phylogenetic analysis

Sections/series	Species	Total size (bp)	PCG size (bp)	tRNA size (bp)	rRNA size (bp)	CR size (bp)	GenBank
Subgenus *Cellia*							
Myzomyia	*An. aconitus*	15,359	11,224	1472	2114	519	NC039540
	*An. culicifacies*	15,364	11,194	1474	2121	535	NC028216
	*An. culicifacies* B	15,330	11,230	1474	2114	498	NC027502
	*An. funestus*	15,356	11,231	1477	2121	519	NC038158
	*An. minimus*	15,411	11,194	1476	2117	546	NC028221
Neocellia	*An. maculatus*	14,850	11,188	1479	2108	N/A	NC028218
	*An. splendidus*	15,362	11,224	1477	2121	510	NC039397
	*An. stephensi*	15,387	11,190	1477	2117	551	NC028223
Neomyzomyia	*An. cracens*	15,412	11,224	1482	2123	576	NC020768
	*An. dirus*	15,406	11,224	1478	2124	568	NC036263
	*An. farauti* 4	15,412	11,224	1482	2125	576	NC020770
	*An. hinesorum*	15,336	11,224	1479	2123	505	NC020769
	*An. punctulatus*	15,322	11,187	1477	2118	493	NC028222
Pyretophorus	*An. arabiensis*	15,369	11,194	1477	2122	530	NC028212
	*An. christyi*	14,967	11,188	1477	2126	N/A	NC028214
	*An. coluzzii*	15,441	11,194	1478	2124	599	NC028215
	*An. epiroticus*	15,379	11,188	1479	2122	535	NC028217
	*An. gambiae*	15,363	11,230	1479	2125	519	NC002084
	*An. melas*	15,366	11,194	1477	2122	526	NC028219
	*An. merus*	15,365	11,188	1478	2121	525	NC028220
Subgenus *Anopheles*							
Angusticorn/Anopheles	*An. atroparvus*	15,458	11,175	1474	2161	614	NC028213
	*An. eiseni geometricus*	15,696	11,241	1474	2120	860	MF381678
	*An. lindesayi*	15,366	11,225	1475	2123	531	KX961140
	*An. quadrimaculatus* A	15,455	11,220	1473	2115	625	NC000875
Laticorn/Arribalzagia	*An. costai*	15,433	11,241	1473	2122	598	NC037794
	*An. nr. costai*	15,434	11,241	1473	2121	600	NC037821
	*An. fluminensis*	15,429	11,241	1474	2120	594	NC037818
	*An. forattinii*	15,459	11,241	1473	2125	615	NC037813
	*An. Medialis*^a^	15,409	11,241	1475	2121	545	NC037789
	*An. minor*	15,466	11,238	1478	2123	594	NC037802
	*An. peryassui*	15,417	11,241	1474	2120	585	NC037790
Laticorn/Myzorhynchus	*An. coustani*	15,408	11,194	1475	2112	570	MT806097
	*An. nitidus*	15,418	11,168	1476	2122	580	MW401801
	*An. peditaeniatus*	15,416	11,224	1477	2125	575	MT822295
	*An. sinensis*	15,418	11,224	1473	2125	577	MF322628
Subgenus *Nyssorhynchus*							
Albimanus/Oswaldoi	*An. albertoi*	15,385	11,240	1475	2114	558	NC037804
	*An. arthuri*	15,387	11,240	1475	2114	560	NC037806
	*An. benarrochi*	15,387	11,240	1477	2116	556	NC037787
	*An. evansae*	15,382	11,240	1477	2115	553	NC037795
	*An. galvaoi*	15,420	11,240	1477	2150	555	NC037814
	*An. goeldii*	15,391	11,240	1477	2117	560	NC037810
	*An. konderi*	15,395	11,240	1478	2125	555	MF381685
	*An. nuneztovari*	15,393	11,240	1477	2117	562	MF381680
	*An. oswaldoi*	15,380	11,237	1477	2115	554	NC037793
	*An. rangeli*	15,386	11,240	1477	2114	558	NC037786
	*An. rondoni*	15,385	11,240	1477	2113	557	NC037815
	*An. striatus*	15,385	11,240	1476	2115	557	NC037801
	*An. strodei*	15,388	11,240	1475	2115	560	NC037808
	*An. triannulatus*	15,401	11,240	1477	2125	559	NC037800
Argyritarsis/Albitarsis	*An. albitarsis*	15,413	11,216	1477	2119	575	NC020662
	*An. albitarsis* F	15,418	11,216	1479	2121	578	NC030768
	*An. albitarsis* G	15,474	11,216	1480	2125	615	NC030766
	*An. braziliensis*	15,397	11,240	1480	2115	562	NC037791
	*An. nr. braziliensis*	15,413	11,240	1478	2116	578	MF381606
	*An. deaneorum*	15,424	11,216	1476	2121	581	NC020663
	*An. janconnae*	15,425	11,216	1480	2120	575	NC030767
	*An. marajoara*	15,453	11,240	1476	2132	584	NC037788
	*An. oryzalimnetes*	15,422	11,216	1479	2120	581	NC030765
Argyritarsis/Argyritarsis	*An. argyritarsis*	15,403	11,240	1481	2115	579	NC037807
	*An. atacamensis*	15,412	11,241	1476	2122	564	NC037792
	*An. darlingi*	15,386	11,240	1489	2122	554	NC014275
	*An. lanei*	15,396	11,240	1478	2116	567	NC037799
	*An. sawyeri*	15,417	11,240	1477	2116	599	NC037798
Myzorhynchella	*An. antunesi*	15,427	11,242	1475	2118	595	NC037817
	*An. guarani*	15,531	11,241	1473	2119	700	NC037816
	*An. lutzii*	15,341	11,242	1475	2118	509	NC037820
	*An. parvus*	15,444	11,235	1470	2116	617	NC037805
	*An. pristinus*	15,405	11,241	1476	2117	581	NC037824
Subgenus *Kerteszia*							
	*An. bellator*	15,668	11,242	1477	2126	811	NC030249
	*An. cruzii*	15,449	11,230	1478	2116	600	NC024740
	*An. homunculus*	15,739	11,242	1475	2125	886	NC030248
	*An. laneanus*	15,446	11,242	1479	2124	591	NC030250
Subgenus *Stethomyia*							
	*An. kompi*	15,505	11,240	1476	2118	647	NC037827
	*An. nimbus*	15,476	11,240	1467	2121	628	NC037811
Subgenus *Lophopodomyia*							
	*An. gilesi*	15,458	11,244	1465	2108	648	NC037803
	*An. pseudotibiamaculatus*	15,597	11,242	1478	2122	768	NC037829
Outgroup							
	*Cx. pipiens pallens*	15,617	11,228	1482	2138	713	KT851543

^a^*Anopheles medialis* = *Anopheles intermedius*

## Results

### Nucleotide composition and genome organization

**Fig. 1:**
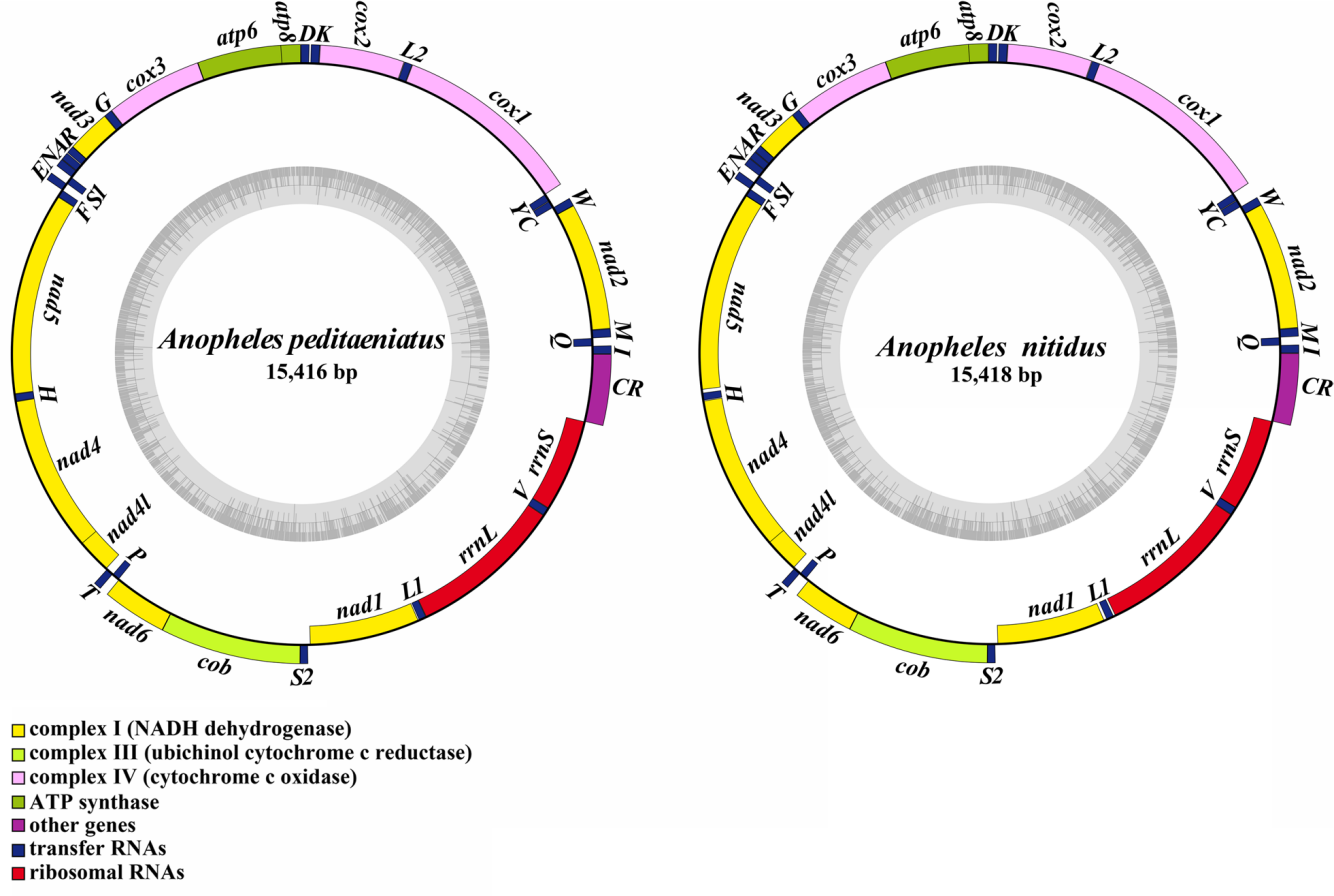
Mitochondrial genome structure of *Anopheles peditaeniatus* and *Anopheles nitidus*

The complete mitogenomes of *An. peditaeniatus* (GenBank: MT822295) and *An. nitidus* (GenBank: MW401801) are both circular genomes with full lengths of 15,416 and 15,418 bp, respectively (Fig. 1). Both are composed of 37 genes (including 13 PCGs, 22 tRNA genes and two rRNA genes) and one control region (CR). There are 22 genes (nine PCGs and 13 tRNAs) located on the majority coding strand (J-strand), while the other 15 genes (four PCGs, nine tRNAs and two rRNAs) on the minority strand (N-strand). Compared with the typical Diptera mitogenome (e.g., *Drosophila yakuba*), both *An. peditaeniatus* and *An. nitidus* have a “*trnR*-*trnA*” rearrangement. The AT content of the mitogenomes of the two species is high, 78.32% and 78.26%, respectively, with obvious AT bias (Additional file 1: Table S1). The AT-skew of *An. peditaeniatus* (0.0322) is higher than the average AT-skew of mosquito mitogenomes (0.0283), whereas the AT-skew of *An. nitidus* mitogenome (0.0266) is lower than the mosquito average. GC-skew in *An. peditaeniatus* (−0.1587) and *An. nitidus* (−0.1536) was higher than the average GC-skew value in mosquitoes investigated (−0.16048).

**Fig. 2:**
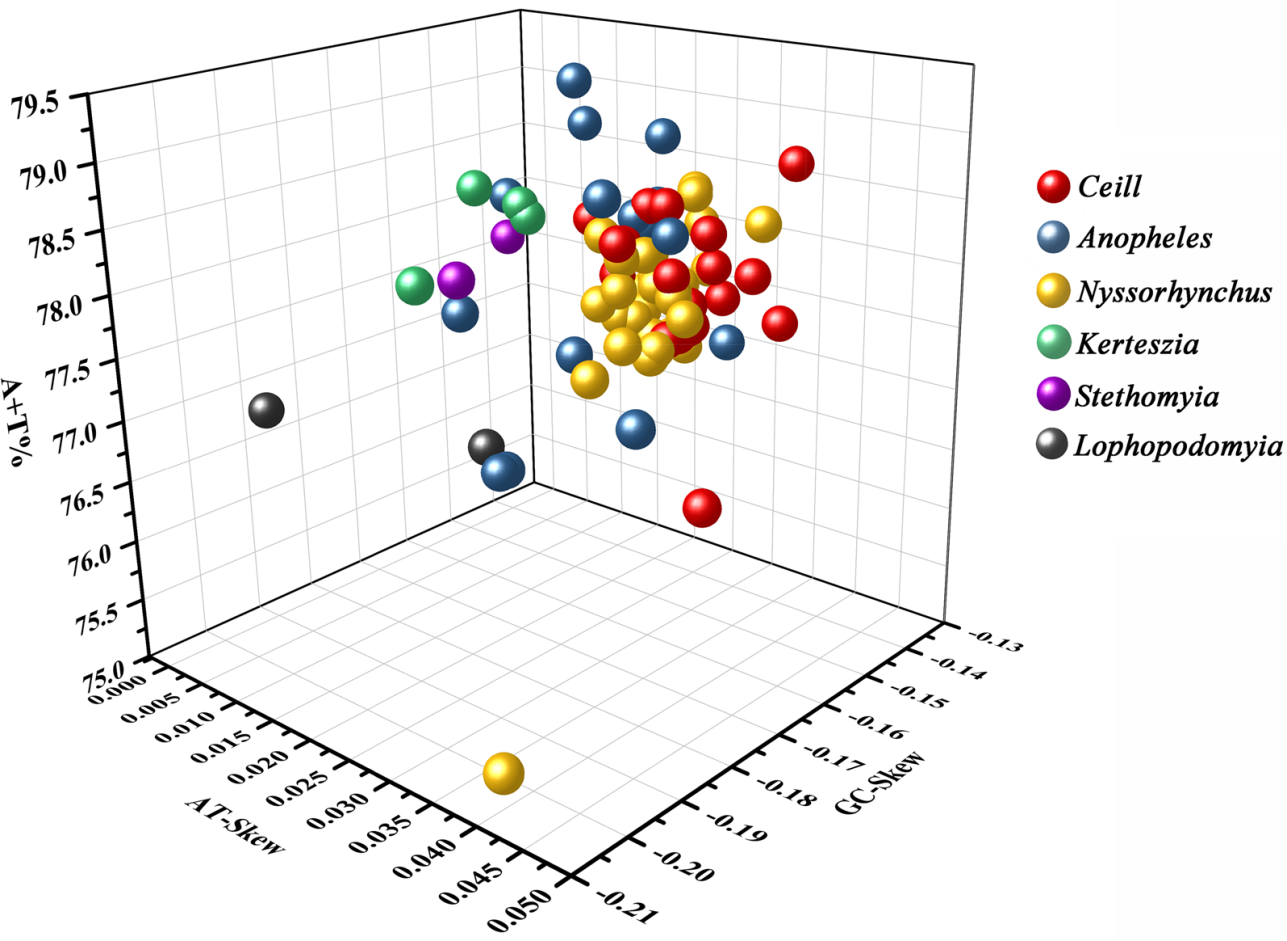
Three-dimensional scatter plot of the AT-skew, GC-skew and AT% of 76 mitochondrial genome sequences of the genus *Anopheles*

The three-dimensional scatter plot of AT content, AT-skew and GC-skew of mitogenomes in the genus *Anopheles* is shown in Fig. 2. AT-skew ranged from 0.005 in *An. gilesi* to 0.043 in *An. christyi*. All mitogenomes display negative GC-skews ranging from −0.207 in *An. parvus* to −0.136 in *An. punctulatus*. Most species of the subgenera *Nyssorhynchus* and *Cellia* have similar AT content and AT/GC-skew (closely distributed in the three-dimensional scatter plot), whereas species in the subgenera *Lophopodomyia, Stethomyia*, *Kerteszia* and *Anopheles* ae widely distributed in the plot for AT content, AT-skew and GC-skew.

### Protein-coding genes

The total nucleotide lengths of the PCGs of *An. peditaeniatus* and *An. nitidus* was 11,223 and 11,168 bp, respectively. In *An. peditaeniatus*, ATN is used as the start codon for all genes except *COX1* and *ND5*, which use TCG and GTG as start codons. In *An. nitidus*, all PCGs initiate with ATN as the start codon, except *COX1*, which uses TCG (Table 2).

**Table 2:** Organization of the *An. peditaeniatus* and *An. nitidus* mitochondrial genomes

Gene	Strand	Position (bp)		Length (bp)		Space(+)/overlap(−)		Start/Stop codon	
		*punctulatus*	*nitidus*	*punctulatus*	*nitidus*	*punctulatus*	*nitidus*	*punctulatus*	*nitidus*
*trnI*	J	1–68	1–68	68	68	0	0		
*trnQ*	N	66–134	66–134	69	69	−3	−3		
*trnM*	J	1134–202	134–202	69	69	−1	−1		
*nad2*	J	203–1228	203–1228	1026	1026	0	0	ATT/TAA	ATT/TAA
*trnW*	J	1227–1295	1227–1295	69	69	−2	−2		
*trnC*	N	1295–1358	1295–1358	64	64	−1	−1		
*trnY*	N	1360–1425	1360–1425	66	66	1	1		
*cox1*	J	1424–2960	1424–2965	1537	1542	−2	−2	TCG/T	TCG/TAA
*trnL2*	J	2961–3026	2961–3026	66	66	0	−5		
*cox2*	J	3028–3712	3028–3712	685	685	1	1	ATG/T	ATG/T
*trnK*	J	3713–3784	3713–3784	72	72	0	0		
*trnD*	J	3797–3865	3797–3865	69	69	12	12		
*atp8*	J	3866–4027	3866–4027	162	162	0	0	ATT/TAA	ATT/TAA
*atp6*	J	4021–4701	4021–4701	681	681	−7	−7	ATG/TAA	ATG/TAA
*cox3*	J	4701–5487	4701–5495	787	795	−1	−1	ATG/T	ATG/TAA
*trnG*	J	5488–5554	5488–5554	67	67	0	−8		
*nad3*	J	5555–5908	5555–5908	354	354	0	0	ATA/TAA	ATA/TAA
*trnR*	J	5907–5970	5907–5970	64	64	−2	−2		
*trnA*	J	5974–6038	5971–6036	65	66	3	0		
*trnN*	J	6039–6105	6037–6103	67	67	0	0		
*trnS1*	N	6106–6172	6104–6170	67	67	0	0		
*trnE*	J	6174–6239	6172–6237	66	66	1	1		
*trnF*	N	6238–6304	6236–6302	67	67	−2	−2		
*nad5*	N	6304–8046	6302–8017	1743	1766	−1	−1	GTG/TAA	ATT/TAA
*trnH*	N	8047–8110	8045–8109	64	65	0	27		
*nad4*	N	8111–9452	8113–9451	1342	1339	0	3	ATG/T	ATG/T
*nad4L*	N	9446–9745	9445–9744	300	300	−7	−7	ATG/TAA	ATG/TAA
*trnT*	J	9752–9816	9751–9815	65	65	6	6		
*trnP*	N	9817–9882	9816–9881	66	66	0	0		
*nad6*	J	9885–10,409	9884–10,408	525	525	2	2	ATT/TAA	ATT/TAA
*cob*	J	10,409–11,545	10,408–11,544	1137	1137	−1	−1	ATG/TAA	ATG/TAA
*trnS2*	J	11,544–11,609	11,543–11,608	66	66	−2	−2		
*nad1*	N	11,628–12,572	11,629–12,573	945	945	18	20	ATT/TAA	ATT/TAA
*trnL1*	N	12,579–12,644	12,580–12,645	66	66	6	6		
*rrnL*	N	12,645–13,972	12,646–13,973	1328	1328	0	0		
*trnV*	N	13,973–14,044	13,974–14,044	72	72	0	0		
*rrnS*	N	14,045–14,841	14,045–14,838	797	794	0	0		
CR		14,842–15,416	14,839–15,418	575	579	0	0		

**Fig. 3:**
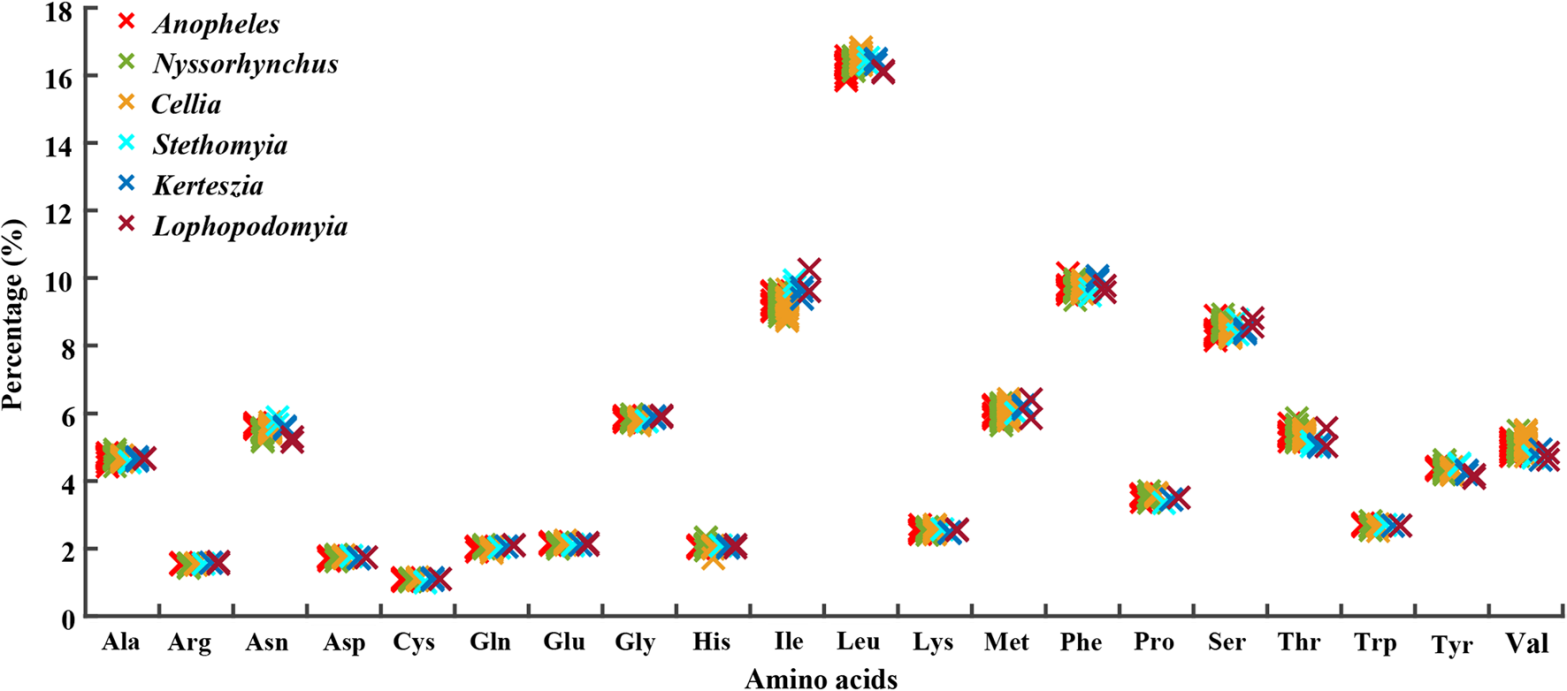
Frequency percentage of each of 20 coded amino acids in 76 mitochondrial genome sequences of the genus *Anopheles*

The RSCU values of mitogenomes in the genus *Anopheles* are presented in Additional file 2: Table S2. *Anopheles* species have different usage frequencies of synonymous codons; UUA is the most frequently used codon, followed by CGA, GGA, GCU. The amino acid Leu has the highest usage percentage for all 76 mitogenomes investigated with an average of 16.37%, followed by Phe (9.69%), Ile (9.31%) and Ser (8.48%), whereas Cys has the lowest percentage (0.99%). The usage percentages of amino acids do not differ significantly between different subgenera (Fig. 3).

**Fig. 4:**
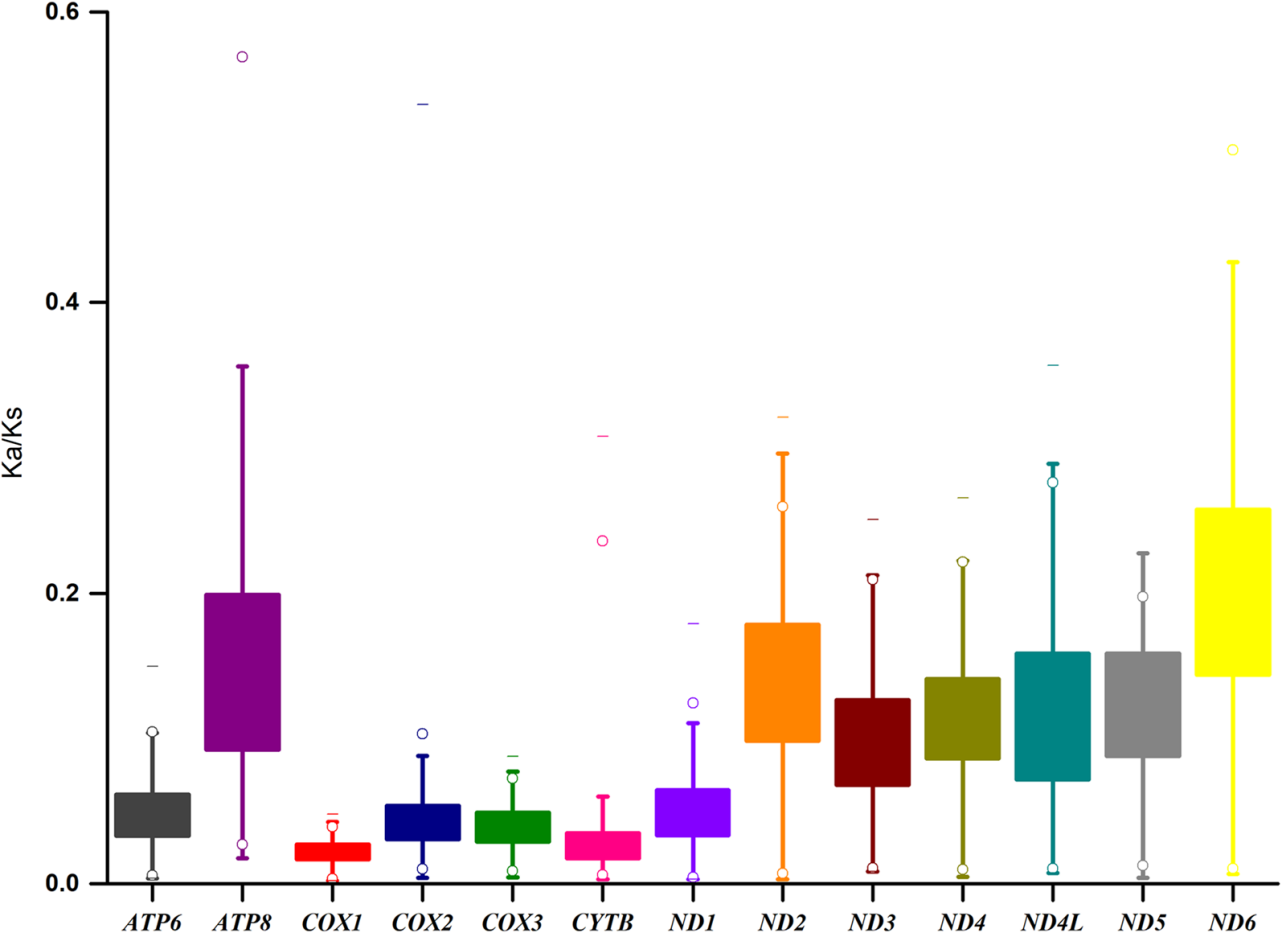
Evolutionary rates of 13 protein-coding genes (PCGs) within 76 mitochondrial genomes of the genus *Anopheles*. Ka: Non-synonymous mutation rate; Ks: Synonymous mutation rate; Ka/Ks: The ratio of non-synonymous mutation rate to synonymous mutation rate. Neutral evolution (Ka/Ks = 1), Purify selection (Ka/Ks < 1), Positive selection (Ka/Ks > 1)

The non-synonymous (Ka) and synonymous (Ks) substitution ratio (Ka/Ks) of the PCGs are shown in Fig. 4. The Ka/Ks ratios are all less than 1, with *ND6* the highest (0.203), followed by six genes (*ATP8*, *ND2*, *ND5*, *ND4L*, *ND4*, *ND3*) with Ka/Ks ratios of 0.098–0.152. Complex IV (*COX1*, *COX2* and *COX3*), Complex III (*CYTB*), *ND1* and *ATP6* have low Ka/Ks ratios with range from 0.022 (*COX1*) to 0.051 (*ND1*). These results imply that all PCGs have experienced purifying selection, especially Complex IV, Complex III, *ND1* and *ATP6*.

### Transfer RNAs, ribosomal RNAs and CR

The total length of tRNAs in *An. peditaeniatus* and *An. nitidus* was 1475 bp and 1476 bp, respectively, while the length of individual tRNAs varies from 64 to 72 bp. All tRNAs can fold into the typical clover-leaf structure of four stems and loops, except for *trnS2* which has lost the dihydrouridine (DHU) arm (Additional file 3: Figure S1). The length of the rRNAs was 2125 bp, with an AT content of 81.36% in *An. punctulatus* and 2122 bp, with an AT content of 81.39% in *An. nitidus*.

The control regions (CRs) of *Anopheles* mitogenomes are located between *rrnS* and *trnI*, with lengths of 575 and 580 bp and AT content of 94.43% and 93.62% in *An. peditaeniatus* and *An. nitidus*, respectively. Six repeat unit types are found in the CRs of *Anopheles* mitogenomes (Additional file 4: Figure S2). All species have 15–27 bp poly-T stretch, located immediately after 140–212 bp of conserved sequence. The poly-T stretch is adjacent to the conserved motif 5′-CCCCTA-3′ in 68 species, whereas this motif was replaced by 5′-ATTGTA-3′ in *An. cracens* and *An. dirus*, and 5′-TTCCCC-3′ in *An. kompi*, *An. nimbus*, *An. gilesi* and *An. pseudotibiamaculatus*. The repeat type is 12–55 bp long and composed of 2–6 repeats, located downstream of the poly-T stretch, and is found in 54 species. The third type ([TA(A)] n stretch) with 22–91 repeats, is found in 36 species. The fourth type is a 12–38 bp region composed of 2–5 repeats adjacent to *trnI* and found in 40 species. The remaining two repeat unit types are found in only a few species; one is a 15–36 bp region located after the second repeat type and found in five species, while the last type is a 108–171 bp region, the longest of the six types and found in only four species.

### Phylogenetic relationships

**Fig. 5:**
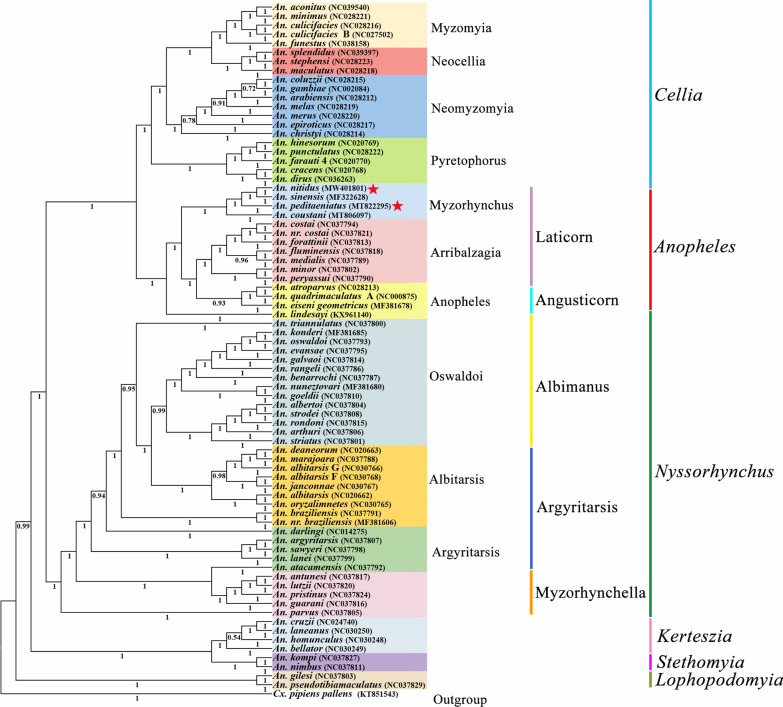
Phylogenetic relationships of 76 mitochondrial genomes of the genus *Anopheles*. The phylogenetic tree was constructed based on nucleotide sequences of 13 protein-coding genes using MrBayes Inference. The numbers at the nodes are Bayesian posterior probabilities. The mitochondrial genomes of two species newly sequenced in this study are indicated by pentagrams. The GenBank accession numbers of the 76 mitochondrial genome sequences are listed in Table 1

**Fig. 6:**
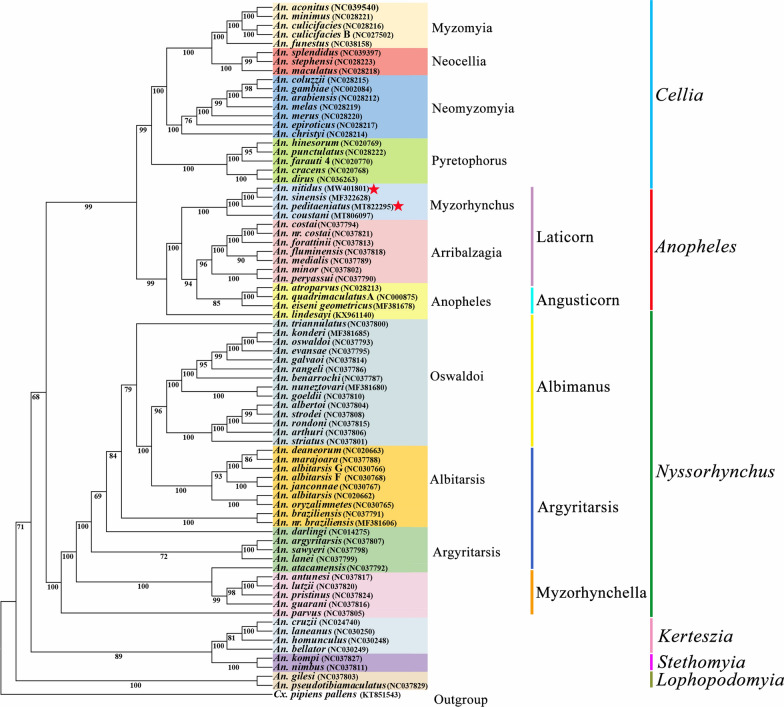
Phylogenetic relationships of 76 *Anopheles* spp. based on mitochondrial genomes. The phylogenetic tree was constructed based on nucleotide sequences of 13 protein-coding genes using maximum likelihood. The numbers at the nodes are bootstrap values. The mitochondrial genomes of two species newly sequenced in this study are indicated by pentagrams. The GenBank accession numbers of the 76 mitochondrial genome sequences are listed in Table 1

Bayesian inference (BI) and maximum-likelihood (ML) analyses produced the same phylogenetic trees at the subgenus level (Figs. 5 and 6). The six subgenera investigated, *Lophopodomyia*, *Stethomyia*, *Kerteszia*, *Nyssorhynchus*, *Anopheles* and *Cellia*, are monophyletic in both analyses, with the posterior probability (pp) = 1 for every subgenus (Fig. 5) and bootstrap values (bv) that range from 99 to 100% in ML analysis (Fig. 6). The subgenus *Lophopodomyia* is sister to remaining five subgenera, the clade of which has support of pp = 0.99 and bv = 71%. The two subgenera *Stethomyia* and *Kerteszia* are sisters (pp = 1 and bv = 89%). The clade of *Nyssorhynchus*, *Anopheles* and *Cellia* was well supported (pp = 1 and bv = 68%). The subgenus *Nyssorhynchus* is sister to the clade *Anopheles* + *Cellia* (pp = 1 and bv = 99%).

In the subgenus *Cellia*, four series investigated, Myzomyia, Neocellia, Pyretophorus and Neomyzomyia, were each monophyletic (pp = 1 and bv = 100%). The series Neomyzomyia was sister to the remaining three series. In the subgenus *Anopheles*, both Angusticorn and Laticorn were polyphyletic, while within section Laticorn both series Arribalzagia (pp = 1 and bv = 96%) and Myzorhynchus (pp = 1 and bv = 100%) were monophyletic. In *Nyssorhynchus*, all three sections investigated, Myzorhynchella, Argyritarsis and Albimanus, were polyphyletic, while in section Argyritarsis, both series Argyritarsis and Albitarsis were polyphyletic as well.

## Discussion

### Characteristics of the mitogenome sequences of the genus *Anopheles*

Comparison of mitogenome sequences in the genus *Anopheles* shows that the length variation mainly exists in the CRs, similar to earlier reported mitogenomes in insects [43, 44]. The gene number and the gene composition, codon usage and tRNA secondary structures are similar to other reported mitogenomes of Diptera [22, 45]. However, the *trnR* and *trnA* have a reversed arrangement to form “*trnR*-*trnA*” in comparison to the ancestral insect, as those reported in other genera in Culicidae [21, 45].

The present study identified six repeat unit types in CRs for the first time in *Anopheles* mitogenomes. Among the six types, the poly-T stretch has also been found in other insects, which may involve the identification of the replication origin of mitochondrial DNA (mtDNA) [46]. The conserved sequences in CRs have been reported to be taxon-specific and of evolutionary information, and have been used as important evidence in the inference of phylogenetics in the taxa of the genus *Culex* and *Lutzia* and taxon [47]. However, the evolutionary information carried in the genus *Anopheles* does not seem stable and reliable.

### Phylogenetic relationships

This present study suggests that all six subgenera investigated are monophyletic, and the phylogenetic analysis shows that subgenus *Lophopodomyia* is the sister to all five other subgenera, and the remaining five subgenera are divided into two clades, one including a sister-taxon (*Stethomyia* + *Kerteszia*), and the other consisting of subgenus *Nyssorhynchus* as the sister to a sister-group subgenera *Anopheles* + *Cellia*. A phylogenetic study based on 163 morphological characters for 64 species in the subfamily Anophelinae using the approximate weighting (AW) method showed that the subgenera *Lophopodomyia*, *Stethomyia*, *Kerteszia*, *Nyssorhynchus* and *Cellia* were monophyletic, whereas the subgenus *Anopheles* was polyphyletic. Two subgenera, *Lophopodomyia* and *Stethomyia*, were nested within the subgenus *Anopheles* [12]. A later morphology-based phylogenetic analysis, which used 167 characters for 66 species in the Anophelinae analyzed with both the equal weighting (EW) and implied weighting (IW) methods, found the same results as described above [14]. All analyses from these three methods showed that the subgenera *Nyssorhynchus* and *Kerteszia* were sister-taxa, while the AW and EW methods suggested that the *Nyssorhynchus* + *Kerteszia* was sister-group to subgenus *Cellia* + subgenera *Lophopodomyia*, *Stethomyia* and *Anopheles*, and the IW method found a clade comprising the sister-taxon (*Nyssorhynchus* + *Kerteszia*) and subgenus *Cellia*, and the this clade was sister-group to three subgenera *Lophopodomyia*, *Stethomyia* and *Anopheles*. In contrast, a molecular-based phylogenetic analysis, using *COI*, *COII* and 5.8S rRNA for 47 species of *Anopheles* and using the ML method, supported the monophyly of the subgenera *Stethomyia*, *Kerteszia*, *Nyssorhynchus*, *Anopheles* and *Cellia*, and this study suggested the subgenus *Anopheles* was sister-group to all other subgenera, and placed the subgenus *Cellia* as a sister-group to a clade which comprised subgenus *Nyssorhynchus* and a sister-taxon (*Stethomyia* + *Kerteszia*) [48]. A recent study of amino acid sequences of 1085 single-copy orthologous genes from 18 species in the subgenera *Nyssorhynchus*, *Anopheles* and *Cellia* analyzed with the ML method found that all three subgenera were monophyletic, and showed that the subgenus *Nyssorhynchus* was sister to a sister-taxon (*Anopheles* + *Cellia*) [49]. Our prior study using mitogenome PCG nucleotide sequences from 50 species in Culicidae with the ML and BI methods showed that the subgenera *Nyssorhynchus*, *Anopheles* and *Cellia* were monophyletic, with the sister relationship between subgenus *Nyssorhynchus* and a sister-taxon (*Anopheles* + *Cellia*) [16].

All six *Anopheles* subgenera included in the comprehensive phylogenetic analyses discussed above were suggested to be monophyletic except for the subgenus *Anopheles*, which was recognized as polyphyletic in both morphology-based inferences, while it was monophyletic in the three molecular-based inferences. Importantly, the study based on 18 whole nuclear genomes showed that the subgenus *Anopheles* was monophyletic [49]. The present study supported the monophyly of all six subgenera. Studies based on 18 whole nuclear genomes [50] and 50 whole mitogenomes [16] both suggested that the subgenus *Nyssorhynchus* was sister to the sister-group (*Anopheles* + *Cellia*), as does the present study. A recent study based on *COI*, *COII* and 5.8S rRNA found that the subgenera *Stethomyia* and *Kerteszia* were sisters [48], as in the present study. The subgenus *Lophopodomyia* was grouped with the subgenera *Anopheles* and *Stethomyia* in both morphology-based studies [12, 14], whereas it has not previously been included in molecular-based studies [16, 48, 49]. The current study found that *Lophopodomyia* was sister to the other five subgenera. In general, the phylogenetic relationships inferred from morphology and those based on molecular data are quite different, and further studies are needed including more species and data to elucidate relationships among subgenera.

Within the subgenus *Cellia*, the four series Neomyzomyia, Pyretophorus, Neocellia and Myzomyia that were investigated all appear to be monophyletic (pp = 1 and bv = 100% for their clades), and Neomyzomyia was a sister-group to all other three series, and Pyretophorus was a sister to the sister-taxon (Neocellia + Myzomyia). The current results are consistent with those from our earlier study, those also based on whole mitogenomes [16], and almost close to those based on 18S, 28S, *COI* and *COII* data in both taxon monophyly and relationships [50]. However, the early morphology-based study found all four series to be paraphyletic [12]. These suggest that results stemmed from molecular and morphology are often conflicting as discussed above.

Within the subgenus *Anopheles*, the two sections Angusticorn (from which only series Anopheles was included) and Laticorn (two series Myzorhynchus and Arribalzagia included) are both polyphyletic. The series Myzorhynchus and Arribalzagia are both monophyletic (pp = 1 and bv ≥ 96% for their clades), while if *An. lindesayi* were excluded, the series Anopheles would also be monophyletic (pp = 0.93 and bv = 85%), with the sister relationship between Anopheles and a sister-taxon (Myzorhynchus + Arribalzagia). Analysis of *COI*, *COII* and 5.8S rRNA suggested that the sections Laticorn and Angusticorn and the series Anopheles and Myzorhynchus were polyphyletic. In one morphology-based study, the sections Laticorn and Angusticorn and the series Myzorhynchus and Anopheles were paraphyletic [12]. The other morphology study found section Laticorn and the series Arribalzagia and Myzorhynchus to be monophyletic, while section Angusticorn and the series Anopheles were polyphyletic [14]. All of these studies suggested that section Angusticorn and series Anopheles were polyphyletic, and most studies found the section Laticorn to be polyphyletic, whereas series Arribalzagia was always monophyletic while series Myzorhynchus may be monophyletic.

Within the subgenus *Nyssorhynchus*, three sections, Myzorhynchella, Argyritarsis and Albimanus, were investigated, and the subdivisions in all three sections all appear to be polyphyletic or paraphyletic. A morphology study suggested that sections Albimanus, Argyritarsis and Myzorhynchella were paraphyletic [12]. Two molecular studies found the three sections to be not monophyletic, [51, 52]. All four studies demonstrate that the taxonomy and phylogenetics of *Nyssorhynchus* are quite conflicted, with more study necessary to reconstruct their taxonomic system.

## Conclusions

This study analyzed the complete mitogenomes of *An. peditaeniatus* and *An. nitidus* and investigated phylogenetic relationships among 76 species in the genus *Anopheles*. These mitogenomes have the same general characteristics found in earlier reports from insects; however, the *trnR* and *trnA* are reversed in comparison to other Diptera mitogenomes, as has been reported in other genera in the Culicidae. Genome variations mainly occur in the CR regions, which range in length from 493 to 886 bp and have six repeat regions, identified for the first time. The subgenera *Lophopodomyia*, *Stethomyia*, *Kerteszia*, *Nyssorhynchus*, *Anopheles* and *Cellia* were all found to be monophyletic and showed a new phylogenetic relationship among the six subgenera investigated. Four series Neomyzomyia, Pyretophorus, Neocellia and Myzomyia in the subgenus *Cellia*, were found to be monophyletic, as were the series Arribalzagia and Myzorhynchus in the subgenus *Anopheles*, while the series *Anopheles* and three sections in *Nyssorhynchus*, Myzorhynchella, Argyritarsis and Albimanus, and their subdivisions were polyphyletic or paraphyletic. Further studies of more mosquito species are needed to elucidate the phylogenetic relationships in the genus *Anopheles*.

## Supplementary Information

**Additional file 1: Table S1.** Composition and skewness of 76 mitochondrial genomes of the genus *Anopheles*.

**Additional file 2: Table S2.** Relative synonymous codon usage (RSCU) in 76 mitochondrial genomes of the genus *Anopheles*.

**Additional file 3: Figure S1.** Predicted secondary structures of 22 tRNAs in the mitochondrial genomes of *An. peditaeniatus* (**a**), *An. nitidus* (**b**).

**Additional file 4: Figure S2.** Repeat unit types of the CRs in the 74 mitochondrial genomes of the genus *Anopheles*. The pentagrams denote poly-T stretch, and the location and copy number of other repeat types are shown by colored dots: orange represents the second type; purple represents the third type ([TA(A)] n Stretch); blue represents the fourth type; pink represents the fifth type; green represents the sixth type. Non-repeat regions are indicated by colored box.

## Data Availability

All data are available as tables and figures in the main document and its additional files. The GenBank accession numbers for the two mitogenomes produced in the present study are MW401801 and MT822295.

## Abbreviations

Mitogenome: Mitochondrial genome
PCGs: Protein-coding genes
rRNAs: Ribosomal RNA genes
tRNAs: Transfer RNA genes
CR: Control region
RSCU: Relative synonymous codon usage
BI: Bayesian inference
ML: Maximum likelihood
