# Supplementary material for: Complete mitogenomes of Anopheles peditaeniatus and Anopheles nitidus and phylogenetic relationships within the genus Anopheles inferred from mitogenomes
Source: Parasit Vectors. 2021 Sep 6;14:452. doi: 10.1186/s13071-021-04963-4 (PMC8420037; doi:10.1186/s13071-021-04963-4)
Supplement: Supplementary file 1 — Additional file 1: Table S1. Composition and skewness of 76 mitochondrial genomes of the genus Anopheles. [file 13071_2021_4963_MOESM1_ESM.doc]

Table S1. Composition and skewness of 76 species of mtgenomes in the genus *Anopheles*.

| **Sections/Series** | **Species** | **A%** | **T%** | **G%** | **C%** | **A+T%** | **AT-Skew** | **GC-Skew** |
| --- | --- | --- | --- | --- | --- | --- | --- | --- |
| Subgenus *Cellia* |  |  |  |  |  |  |  |  |
| /Myzomyia | *An. aconitus* | 39.94 | 38.31 | 9.19 | 12.56 | 78.25 | 0.0208 | -0.1549 |
|  | *An. culicifacies* | 40.28 | 37.74 | 9.24 | 12.74 | 78.02 | 0.0326 | -0.1592 |
|  | *An. culicifacies* B | 40.37 | 38.08 | 9.12 | 12.44 | 78.45 | 0.0292 | -0.1540 |
|  | *An. funestus* | 40.28 | 38.00 | 9.08 | 12.64 | 78.28 | 0.0291 | -0.1639 |
|  | *An. minimus* | 40.29 | 38.14 | 9.12 | 12.45 | 78.43 | 0.0274 | -0.1544 |
| /Neocellia | *An. maculatus* | 40.24 | 37.31 | 9.59 | 12.86 | 77.55 | 0.0378 | -0.1457 |
|  | *An. splendidus* | 40.18 | 37.74 | 9.41 | 12.67 | 77.92 | 0.0313 | -0.1476 |
|  | *An. stephensi* | 40.40 | 37.85 | 9.23 | 12.52 | 78.25 | 0.0326 | -0.1513 |
| /Neomyzomyia | *An. cracens* | 40.00 | 37.94 | 9.27 | 12.78 | 77.94 | 0.0264 | -0.1592 |
|  | *An. dirus* | 40.12 | 38.05 | 9.19 | 12.64 | 78.17 | 0.0265 | -0.1580 |
|  | *An. farauti* 4 | 40.12 | 37.78 | 9.32 | 12.79 | 77.89 | 0.0300 | -0.1569 |
|  | *An. hinesorum* | 40.41 | 37.55 | 9.37 | 12.67 | 77.96 | 0.0367 | -0.1497 |
|  | *An. punctulatus* | 40.71 | 37.95 | 9.21 | 12.13 | 78.66 | 0.0351 | -0.1368 |
| /Pyretophorus | *An. arabiensis* | 40.08 | 37.51 | 9.45 | 12.96 | 77.59 | 0.0331 | -0.1566 |
|  | *An. christyi* | 40.01 | 36.68 | 9.63 | 13.67 | 76.69 | 0.0434 | -0.1734 |
|  | *An. coluzzii* | 40.12 | 37.61 | 9.41 | 12.86 | 77.73 | 0.0323 | -0.1549 |
|  | *An. epiroticus* | 40.06 | 37.60 | 9.53 | 12.80 | 77.66 | 0.0317 | -0.1464 |
|  | *An. gambiae* | 40.03 | 37.53 | 9.49 | 12.95 | 77.56 | 0.0322 | -0.1542 |
|  | *An. melas* | 40.06 | 37.50 | 9.44 | 13.00 | 77.56 | 0.0330 | -0.1586 |
|  | *An. merus* | 40.10 | 37.49 | 9.39 | 13.02 | 77.59 | 0.0336 | -0.1620 |
| Subgenus *Anopheles* |  |  |  |  |  |  |  |  |
| Angusticorn/Anopheles | *An. atroparvus* | 39.98 | 37.39 | 9.63 | 13.00 | 77.37 | 0.0335 | -0.1489 |
|  | *An. eiseni geometricus* | 40.30 | 38.95 | 8.80 | 11.94 | 79.26 | 0.0170 | -0.1514 |
|  | *An. lindesayi* | 40.43 | 38.44 | 9.01 | 12.12 | 78.87 | 0.0252 | -0.1472 |
|  | *An. quadrimaculatus* A | 40.25 | 37.11 | 9.27 | 13.37 | 77.36 | 0.0406 | -0.1811 |
| Laticorn/Arribalzagia | *An. costai* | 39.44 | 37.35 | 9.48 | 13.73 | 76.79 | 0.0272 | -0.1831 |
|  | *An. nr. costai* | 39.50 | 37.34 | 9.43 | 13.73 | 76.84 | 0.0281 | -0.1857 |
|  | *An. fluminensis* | 39.77 | 38.65 | 9.05 | 12.53 | 78.42 | 0.0143 | -0.1613 |
|  | *An. forattinii* | 39.74 | 38.11 | 9.07 | 13.08 | 77.85 | 0.0209 | -0.1810 |
|  | *An. intermedius* | 40.02 | 38.83 | 9.03 | 12.12 | 78.85 | 0.0151 | -0.1461 |
|  | *An. minor* | 40.58 | 38.14 | 8.83 | 12.45 | 78.72 | 0.0310 | -0.1701 |
|  | *An. peryassui* | 39.83 | 37.67 | 9.33 | 13.17 | 77.50 | 0.0279 | -0.1707 |
| Laticorn/Myzorhynchus | *An. coustani* | 40.17 | 38.18 | 9.22 | 12.44 | 78.35 | 0.0254 | -0.1487 |
|  | *An. nitidus* | 40.17 | 38.09 | 9.20 | 12.54 | 78.26 | 0.0266 | -0.1536 |
|  | *An. peditaeniatus* | 40.42 | 37.90 | 9.12 | 12.56 | 78.32 | 0.0322 | -0.1587 |
|  | *An. sinensis* | 40.20 | 38.14 | 9.15 | 12.51 | 78.34 | 0.0263 | -0.1551 |
| Subgenus *Nyssorhynchus* |  |  |  |  |  |  |  |  |
| Albimanus/Oswaldoi | *An. albertoi* | 39.94 | 37.73 | 9.41 | 12.93 | 77.67 | 0.0285 | -0.1576 |
|  | *An. arthuri* | 39.86 | 37.57 | 9.55 | 13.02 | 77.43 | 0.0296 | -0.1537 |
|  | *An. benarrochi* | 40.07 | 37.46 | 9.24 | 13.23 | 77.53 | 0.0337 | -0.1776 |
|  | *An. evansae* | 40.07 | 37.97 | 9.30 | 12.66 | 78.04 | 0.0269 | -0.1530 |
|  | *An. galvaoi* | 39.94 | 37.58 | 9.45 | 13.03 | 77.52 | 0.0304 | -0.1593 |
|  | *An. goeldii* | 40.30 | 37.51 | 9.29 | 12.90 | 77.80 | 0.0359 | -0.1627 |
|  | *An. konderi* | 40.21 | 37.92 | 9.20 | 12.67 | 78.13 | 0.0293 | -0.1587 |
|  | *An. nuneztovari* | 40.19 | 37.65 | 9.32 | 12.84 | 77.85 | 0.0326 | -0.1588 |
|  | *An. oswaldoi* | 40.18 | 37.95 | 9.16 | 12.70 | 78.13 | 0.0285 | -0.1619 |
|  | *An. rangeli* | 40.19 | 37.52 | 9.22 | 13.06 | 77.71 | 0.0344 | -0.1724 |
|  | *An. rondoni* | 39.89 | 37.63 | 9.46 | 13.02 | 77.52 | 0.0292 | -0.1584 |
|  | *An. striatus* | 40.05 | 37.45 | 9.39 | 13.11 | 77.50 | 0.0335 | -0.1653 |
|  | *An. strodei* | 39.95 | 37.70 | 9.38 | 12.96 | 77.65 | 0.0290 | -0.1603 |
|  | *An. triannulatus* | 40.06 | 37.78 | 9.47 | 12.68 | 77.84 | 0.0293 | -0.1449 |
| Argyritarsis/Albitarsis | *An. albitarsis* | 39.92 | 37.75 | 9.36 | 12.97 | 77.67 | 0.0279 | -0.1617 |
|  | *An. albitarsis* F | 40.08 | 37.83 | 9.24 | 12.85 | 77.91 | 0.0289 | -0.1634 |
|  | *An. albitarsis* G | 39.97 | 37.78 | 9.47 | 12.78 | 77.75 | 0.0282 | -0.1488 |
|  | *An. braziliensis* | 40.15 | 37.70 | 9.34 | 12.81 | 77.85 | 0.0315 | -0.1567 |
|  | *An. nr. braziliensis* | 40.10 | 37.47 | 9.37 | 13.07 | 77.56 | 0.0339 | -0.1649 |
|  | *An. deaneorum* | 39.91 | 37.84 | 9.42 | 12.83 | 77.75 | 0.0266 | -0.1533 |
|  | *An. janconnae* | 39.88 | 37.74 | 9.43 | 12.95 | 77.62 | 0.0276 | -0.1573 |
|  | *An. marajoara* | 39.98 | 37.79 | 9.42 | 12.81 | 77.77 | 0.0282 | -0.1525 |
|  | *An. oryzalimnetes* | 39.95 | 37.84 | 9.28 | 12.93 | 77.79 | 0.0271 | -0.1643 |
| Argyritarsis/Argyritarsis | *An. argyritarsis* | 40.10 | 37.78 | 9.32 | 12.80 | 77.88 | 0.0298 | -0.1573 |
|  | *An. atacamensis* | 40.61 | 37.73 | 9.23 | 12.43 | 78.34 | 0.0368 | -0.1477 |
|  | *An. darlingi* | 40.22 | 37.98 | 9.32 | 12.48 | 78.20 | 0.0286 | -0.1450 |
|  | *An. lanei* | 40.06 | 37.83 | 9.34 | 12.77 | 77.89 | 0.0286 | -0.1551 |
|  | *An. sawyeri* | 40.38 | 38.09 | 9.17 | 12.35 | 78.47 | 0.0292 | -0.1478 |
| Myzorhynchella/ | *An. antunesi* | 39.76 | 37.84 | 9.50 | 12.89 | 77.60 | 0.0247 | -0.1514 |
|  | *An. guarani* | 40.27 | 38.16 | 9.24 | 12.33 | 78.43 | 0.0269 | -0.1433 |
|  | *An. lutzii* | 40.12 | 38.13 | 9.13 | 12.62 | 78.25 | 0.0254 | -0.1605 |
|  | *An. parvus* | 39.28 | 36.13 | 9.74 | 14.85 | 75.41 | 0.0418 | -0.2078 |
|  | *An. pristinus* | 39.77 | 37.51 | 9.66 | 13.07 | 77.28 | 0.0292 | -0.1500 |
| Subgenus *Kerteszia* |  |  |  |  |  |  |  |  |
|  | *An. bellator* | 39.92 | 38.26 | 8.85 | 12.98 | 78.18 | 0.0212 | -0.1892 |
|  | *An. cruzii* | 39.93 | 38.51 | 9.00 | 12.55 | 78.44 | 0.0181 | -0.1647 |
|  | *An. homunculus* | 39.87 | 38.70 | 8.91 | 12.52 | 78.57 | 0.0149 | -0.1685 |
|  | *An. laneanus* | 39.91 | 38.44 | 9.05 | 12.61 | 78.35 | 0.0188 | -0.1644 |
| Subgenus *Stethomyia* |  |  |  |  |  |  |  |  |
|  | *An. kompi* | 39.89 | 38.23 | 8.95 | 12.93 | 78.12 | 0.0212 | -0.1819 |
|  | *An. nimbus* | 39.49 | 38.53 | 9.25 | 12.73 | 78.02 | 0.0123 | -0.1583 |
| Subgenus *Lophopodomyia* |  |  |  |  |  |  |  |  |
|  | *An. gilesi* | 38.70 | 38.31 | 9.25 | 13.74 | 77.01 | 0.0051 | -0.1953 |
|  | *An. pseudotibiamaculatus* | 39.30 | 37.53 | 9.50 | 13.68 | 76.83 | 0.0230 | -0.1803 |
