# Supplementary material for: Complete mitogenomes of Anopheles peditaeniatus and Anopheles nitidus and phylogenetic relationships within the genus Anopheles inferred from mitogenomes
Source: Parasit Vectors. 2021 Sep 6;14:452. doi: 10.1186/s13071-021-04963-4 (PMC8420037; doi:10.1186/s13071-021-04963-4)
Supplement: Supplementary file 3 — Additional file 3: Figure S1. Predicted secondary structures of 22 tRNAs in the mitochondrial genomes of An. peditaeniatus (a), An. nitidus (b). [file 13071_2021_4963_MOESM3_ESM.doc]

**Figure S1.** **Predicted secondary structures for 22 tRNAs in *An. peditaeniatus* (A), *An. nitidus* (B) mtgenomes.**


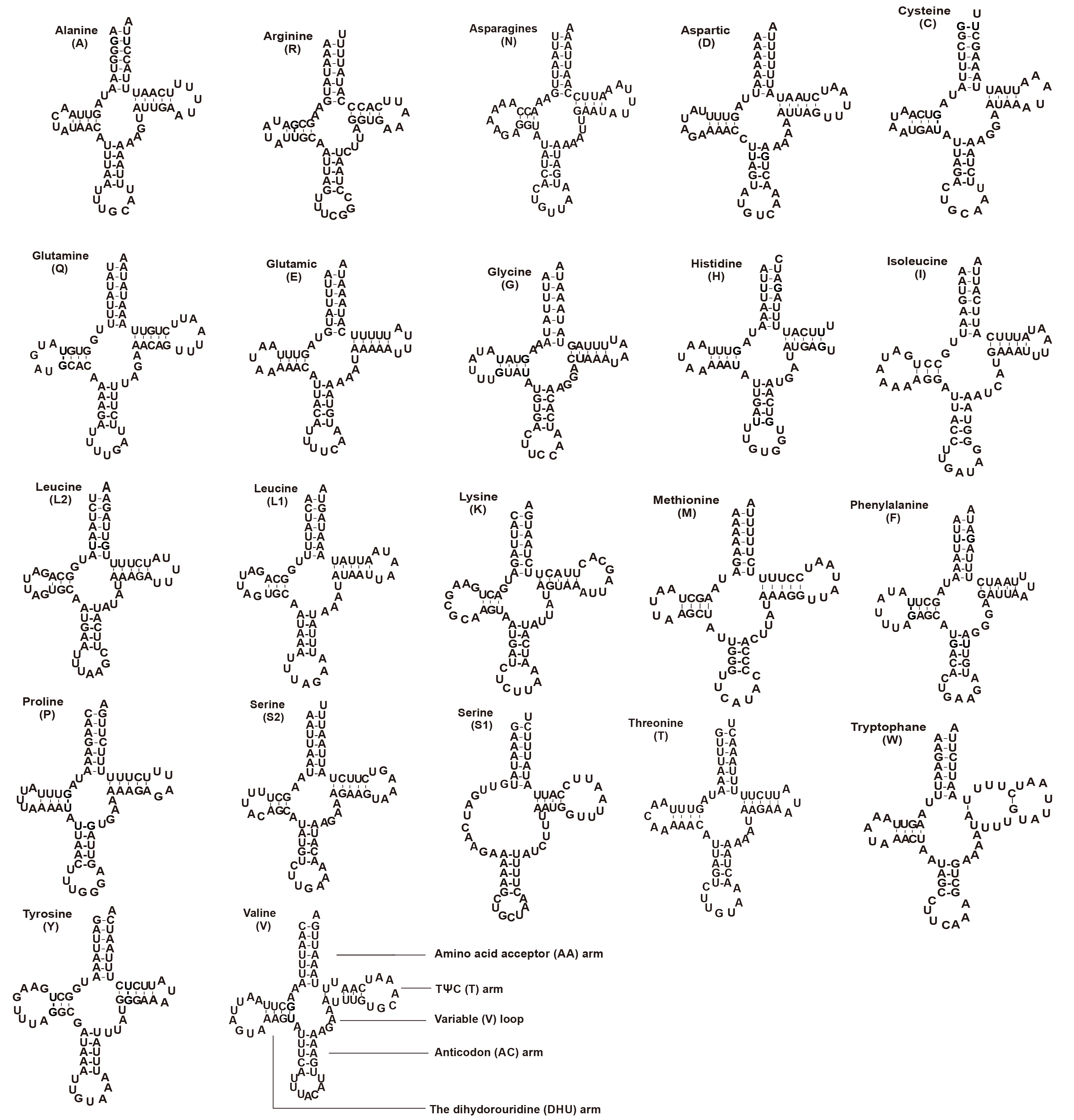
**A**


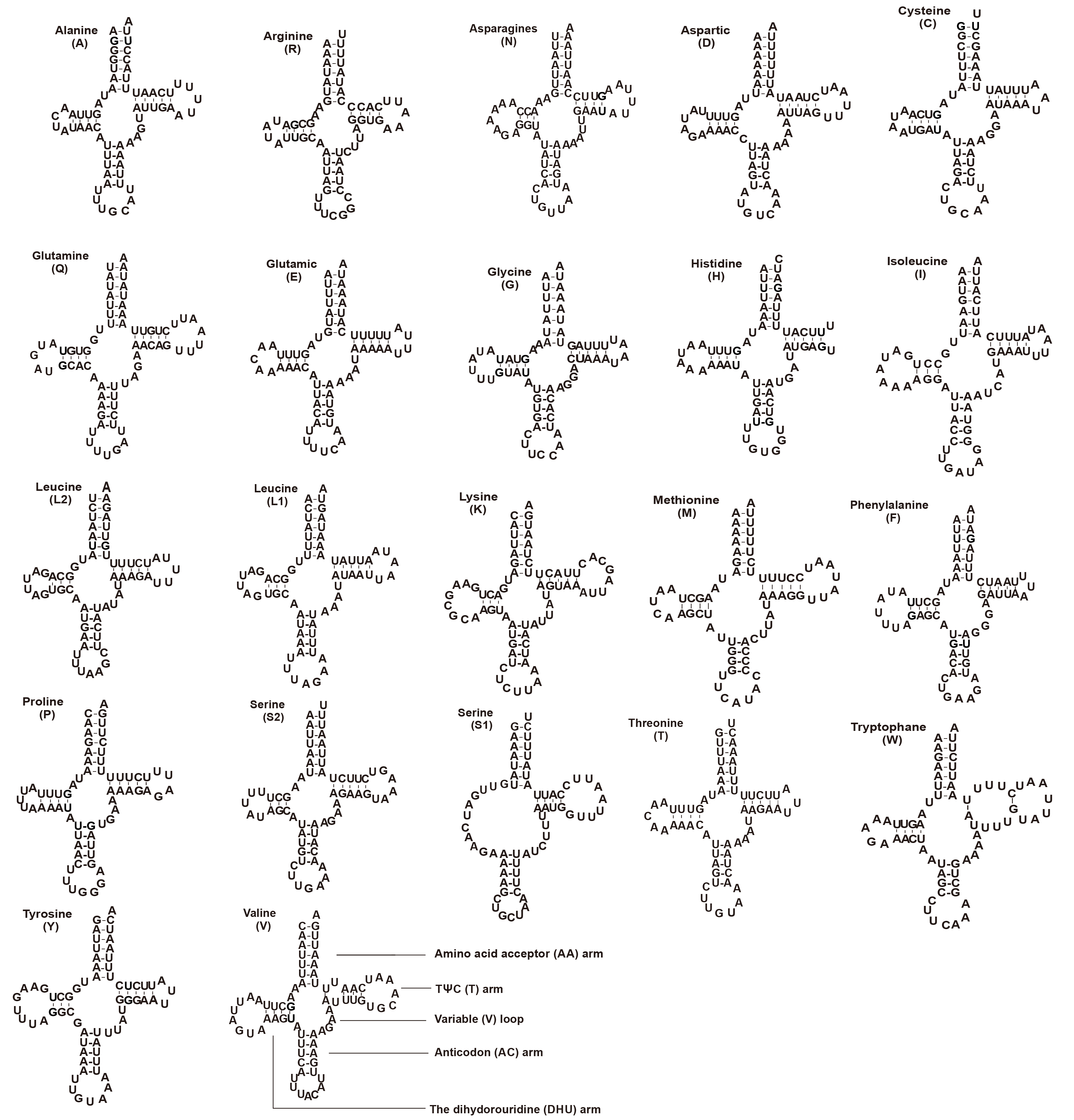
B
