# Supplementary material for: Complete mitogenomes of Anopheles peditaeniatus and Anopheles nitidus and phylogenetic relationships within the genus Anopheles inferred from mitogenomes
Source: Parasit Vectors. 2021 Sep 6;14:452. doi: 10.1186/s13071-021-04963-4 (PMC8420037; doi:10.1186/s13071-021-04963-4)

**Figure S2. Repeat unit types of the CRs in the 74 species of mtgenomes in *Anopheles*.** The pentagrams denote poly-T Stretch, and the location and copy number of other repeat types are shown by colored dots: orange represents the second type; purple represents the third type ([TA(A)]n Stretch); blue represents the fourth type; pink represents the fifth type; green represents the sixth type. Non-repeat regions are indicated by colored box.


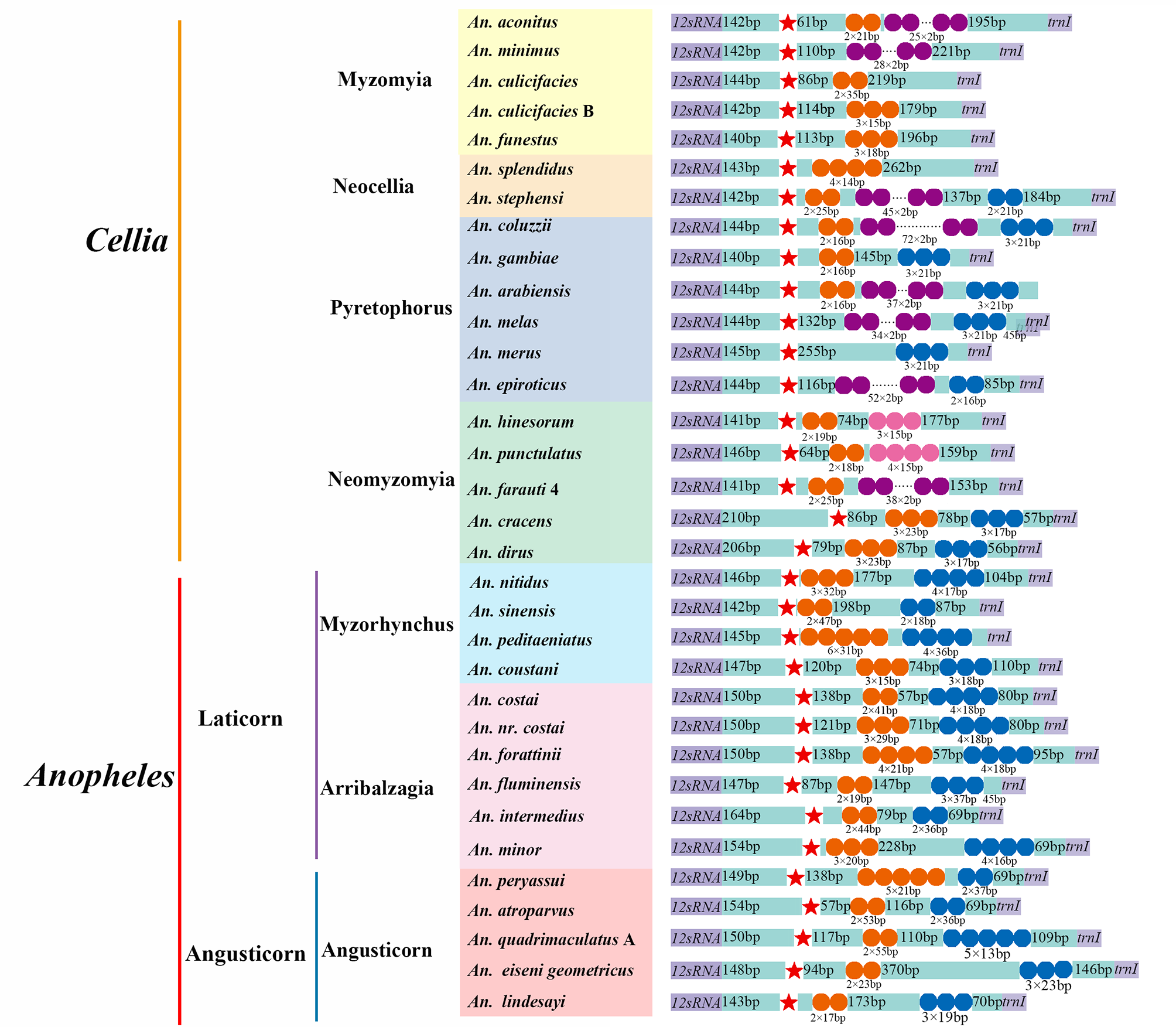


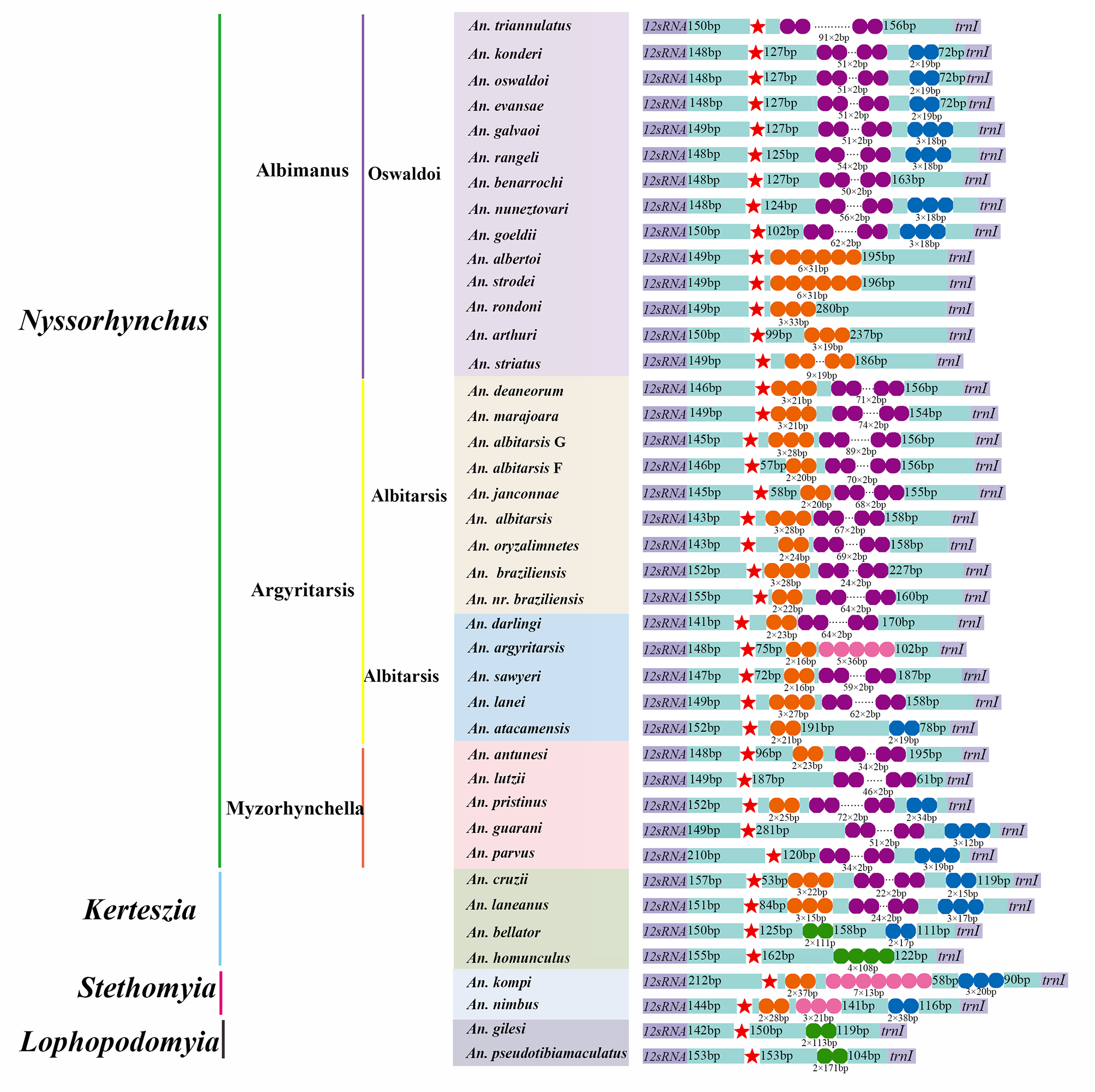

Supplement: Supplementary file 4 — Additional file 4: Figure S2. Repeat unit types of the CRs in the 74 mitochondrial genomes of the genus Anopheles. The pentagrams denote poly-T stretch, and the location and copy number of other repeat types are shown by colored dots: orange represents the second type; purple represents the third type ([TA(A)] n Stretch); blue represents the fourth type; pink represents the fifth type; green represents the sixth type. Non-repeat regions are indicated by colored box. [file 13071_2021_4963_MOESM4_ESM.doc]
